# PEDOT:PSS as a Bio-Solid Electrolyte Interphase for Neural Interfaces: From Molecular Design to Interfacial Intelligence

**DOI:** 10.3390/polym18010020

**Published:** 2025-12-21

**Authors:** Zhen Liu, Jia Liu, Peng Zhang, Xinrong Xu

**Affiliations:** 1 Medical Devices Research and Testing Center, South China University of Technology, Guangzhou 510006, China; liuzhen6060@scut.edu.cn (Z.L.);; 2 School of Materials Science and Engineering, Key Laboratory for Polymeric Composite and Functional Materials of Ministry of Education, Sun Yat-sen University, Guangzhou 510275, China

**Keywords:** PEDOT:PSS, SEI, neural interface, hierarchical structure, structure–property correlation, bioelectronics

## Abstract

Poly(3,4-ethylenedioxythiophene):poly(styrenesulfonate) (PEDOT:PSS) has become one of the most influential materials in neural engineering, offering high electrical conductivity, mechanical softness, and stable processing in complex aqueous media. Beyond these well-known merits, recent studies indicate that PEDOT:PSS can be regarded as a bio-solid electrolyte interphase (bio-SEI) that governs the interactions between neural probes and biological tissue. In this framework, PEDOT:PSS functions as a selective and adaptive interphase that mediates ion and electron transport, buffers mechanical mismatch, and mitigates chemical or biological degradation at the device-tissue boundary. This review critically summarizes the progress in molecular design, synthesis, and post-treatment strategies that enhance PEDOT:PSS stability and compatibility within physiological environments. Developments such as polydopamine-assisted adhesion, zwitterionic modification, and hybridization with soft hydrogels have expanded its role from a passive coating to an active, self-regulating interphase that prolongs implant performance. We further discuss how the hierarchical structure of PEDOT:PSS—from its molecular organization to device-level morphology—contributes to long-term electrochemical and biological stability. By treating PEDOT:PSS as an intrinsic bio-SEI rather than a simple conductive coating, this perspective highlights its central role in the development of durable, biocompatible, and intelligent neural interfaces for next-generation implantable electronics.

## 1. Introduction

The development of implantable neural interfaces has transformed the way electrical signals are recorded and modulated within the nervous system [1,2,3,4]. Such technologies underpin deep brain stimulation, brain–machine interfaces, and closed-loop neuromodulation, where stable and precise transduction between electronic and biological domains is essential [5]. However, despite decades of material advances, the long-term reliability of neural probes remains limited by chronic inflammation, mechanical mismatch, and progressive loss of electrochemical performance [6,7]. The interface between the electrode and the surrounding neural tissue-where ionic and electronic currents meet-is the critical bottleneck that determines both signal fidelity and biological safety [1,3,8].

**Figure 1:**
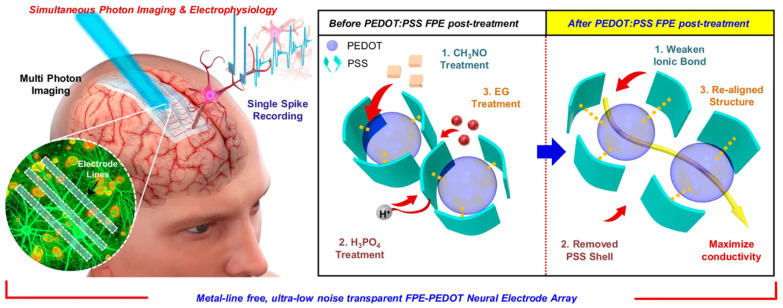
Schematic demonstration of transparent, metal-free PEDOT:PSS neural interfaces. The left part illustrates the device integrated onto the cortical surface, enabling simultaneous two-photon (2P) imaging and high-fidelity electrophysiology. The right part depicts the all-polymer structural layout, where acid-treated PEDOT:PSS serves as both the sensing sites and interconnects. This architecture eliminates metallic photoelectric artifacts and minimizes mechanical mismatch, ensuring stable, chronic bio-integration for multimodal brain mapping. Reproduced from Ref. [12], Springer Nature, 2025.

Among the materials explored to bridge this interface, poly(3,4-ethylenedioxythiophene):poly(styrenesulfonate) (PEDOT:PSS) has emerged as one of the most successful [9,10,11]. Its combination of high electrical conductivity, mechanical compliance, and water processability enables close contact with soft, hydrated tissue while maintaining efficient charge transfer [9]. Since its introduction to neural engineering, PEDOT:PSS has been widely adopted as a coating for metallic electrodes, enhancing charge storage capacity and reducing impedance by several orders of magnitude (Figure 1) [12]. Note: Figure 1 is adapted from a recent experimental study demonstrating secondary-doping enabled PEDOT:PSS neural electrodes for multimodal recording [12]. While the original work focuses on conductivity enhancement through specific solvent and acid treatments, the schematic also captures more general features that are highly relevant to neural biointerfaces, including ion penetration, hydration, and chemically heterogeneous interfacial regions. In this review, the figure is therefore used as a conceptual framework to discuss how such chemically modified PEDOT:PSS surfaces may evolve toward stabilized bio-interphases under physiological conditions.

Yet, as research in neural interfaces matures, PEDOT:PSS is increasingly recognized not only as a conductive film but as a functional interphase-a dynamic layer that governs chemical, electrical, and mechanical exchanges at the device-tissue boundary [9]. A representative example of the translational potential of PEDOT:PSS-based neural interfaces is provided by the integrated brain–machine interface platform reported by Musk et al. [13]. In this work, PEDOT:PSS-coated gold electrodes were implanted chronically in vivo, enabling high-channel-count neural recording with stable electrochemical performance. This study represents a critical validation of PEDOT:PSS not only as a conductive coating, but as a functional interfacial material capable of operating reliably within complex neural tissue environments.

**Figure 2:**
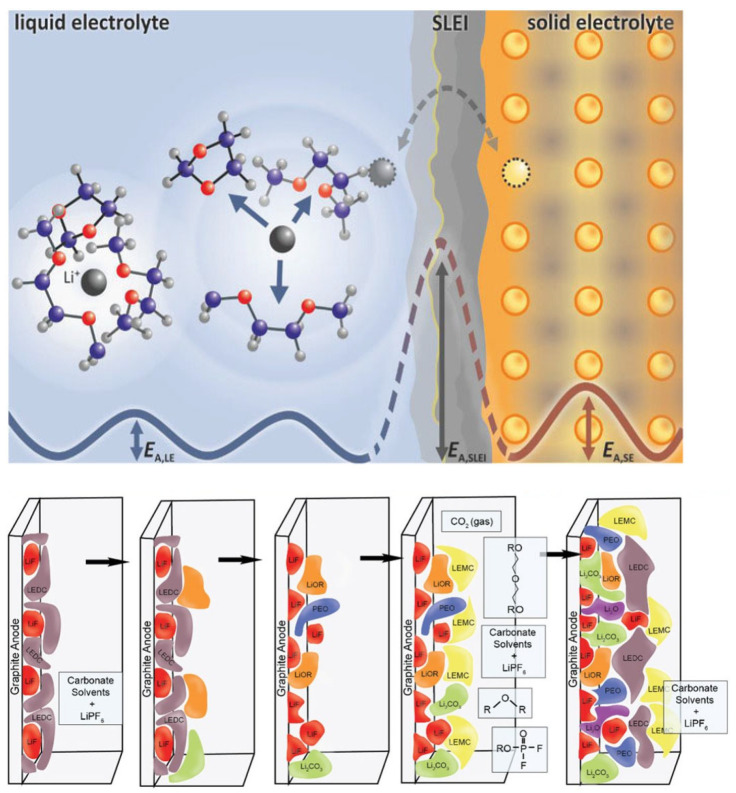
Schematic representation of ion transport mechanisms at the phase boundary between liquid (LE) and solid (SE) electrolytes. The left part illustrates ion migration within the liquid bulk, where ions are surrounded by a coordinated solvation shell. The right part depicts the transition across the heterogeneous phase boundary, highlighting the desolvation process and subsequent transport through the solid/liquid electrolyte interphase (SLEI) into the SE bulk. This model provides a fundamental framework for understanding the energetic barriers and interfacial stability required for developing advanced solid-electrolyte-inspired biointerfaces. llustration of the in situ formation and dynamic evolution of the SEI layer at the liquid electrolyte–solid electrode interface during battery operation, highlighting changes before and after charge/discharge cycling. Reproduced from Ref. [14], John Wiley and Sons, 2020. Reproduced from Ref. [16], John Wiley and Sons, 2023.

This evolving understanding parallels concepts from electrochemical energy storage, particularly the solid–electrolyte interphase (SEI) in lithium-ion batteries (Figure 2) [14,15,16]. In that context, the SEI forms spontaneously at the electrode–electrolyte interface, serving as a thin yet selective barrier that maintains ion transport while preventing further decomposition and preserving system stability [17,18,19,20]. Analogously, PEDOT:PSS can be viewed as a bio-SEI for neural probes-a designed interphase that performs a similar mediating role, maintaining ionic conductivity, preventing harmful reactions, and adapting mechanically to the surrounding tissue [21,22]. This bio-SEI perspective offers a coherent framework to interpret the diverse functions of PEDOT:PSS and to guide its future development toward more durable and biocompatible neural systems.

From a materials standpoint, PEDOT:PSS naturally exhibits several SEI-like characteristics. The PEDOT backbone provides electronic conduction through its conjugated network, while the PSS counterion phase enables ionic transport and processability in water [23,24,25,26]. The resulting mixed ion-electron conduction is key to charge transduction at biological interfaces [9,10,27]. Furthermore, its soft, hydrated morphology minimizes interfacial stress, while its tunable composition allows incorporation of dopants, crosslinkers, and biological macromolecules that control permeability and chemical stability [23,28]. These properties allow PEDOT:PSS to act as a selective and adaptive interface rather than a passive coating.

Despite these advantages, several challenges remain before PEDOT:PSS can fully realize its potential as a bio-SEI. Residual oxidants, dopants, and small molecules from synthesis and post-treatment may leach into surrounding tissue, raising biocompatibility concerns [23,29]. Mechanical degradation, delamination, and loss of conductivity over time also limit chronic implant performance [9,10]. Addressing these issues requires a new design philosophy-one that treats the polymer itself as a self-regulating interphase whose chemistry and structure can be engineered for long-term function within the complex neural environment.

This review aims to consolidate recent progress and provide such a framework. We first revisit the chemical and structural foundations of PEDOT:PSS that underpin its success in flexible and bioelectronic devices. We then discuss how the unique neural environment introduces distinct physicochemical constraints that redefine performance requirements. Building upon this understanding, we outline strategies to design PEDOT:PSS-based bio-SEIs through controlled synthesis, surface modification, and hybrid architectures-that enhance stability, compatibility, and durability of neural probes. Finally, we highlight emerging directions where this concept intersects with self-healing materials, adaptive interfaces, and biointelligent systems. Through this lens, PEDOT:PSS is redefined not merely as a conductive polymer but as the core interphase that enables seamless and sustainable communication between electronics and the nervous system.

## 2. PEDOT:PSS in Chemistry and Flexible Electronics: The Baseline of Success

The remarkable trajectory of PEDOT:PSS began in the field of organic electronics, where its combination of high conductivity, processability, and environmental stability solved long-standing challenges in polymeric conductors [23]. The material’s architecture-a positively charged conjugated polymer (PEDOT) electrostatically balanced by a polyanionic counterion (PSS)-introduced a unique dual-phase morphology that supports simultaneous ionic and electronic transport [9,30]. In PEDOT:PSS, electronic charge transport occurs primarily through the π-conjugated PEDOT-rich domains, while ionic transport is facilitated by the hydrophilic and ion-permeable PSS-rich regions. The microphase-separated morphology, consisting of conductive PEDOT aggregates embedded in a PSS matrix, enables simultaneous transport of electrons (or holes) and ions, a feature that distinguishes PEDOT:PSS from conventional electronic conductors and is central to its bioelectronic functionality. This feature also underpins its use in flexible optoelectronic devices such as organic solar cells, thermoelectric modules, and light-emitting diodes, where intimate contact between electronic and ionic components is essential for efficient charge conversion [23,31,32,33].

**Figure 3:**
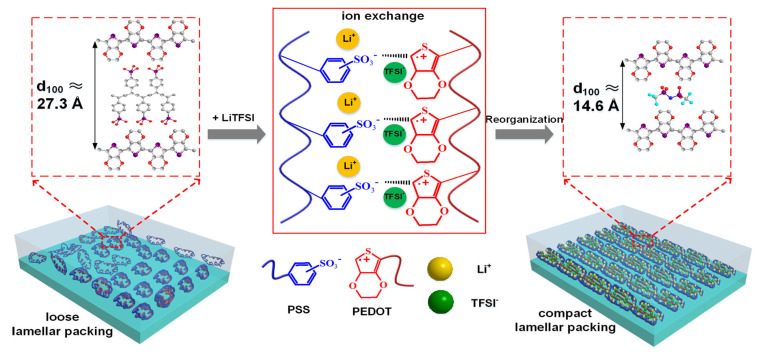
Schematic illustration of the structural evolution of the polymer composite film upon LiTFSI doping, showing loose lamellar packing in pristine PEDOT:PSS (**left**) and compacted lamellar packing in the PEDOT:PSS/LiTFSI composite (**right**). Reproduced from Ref. [30], American Chemical Society, 2021.

**Figure 4:**
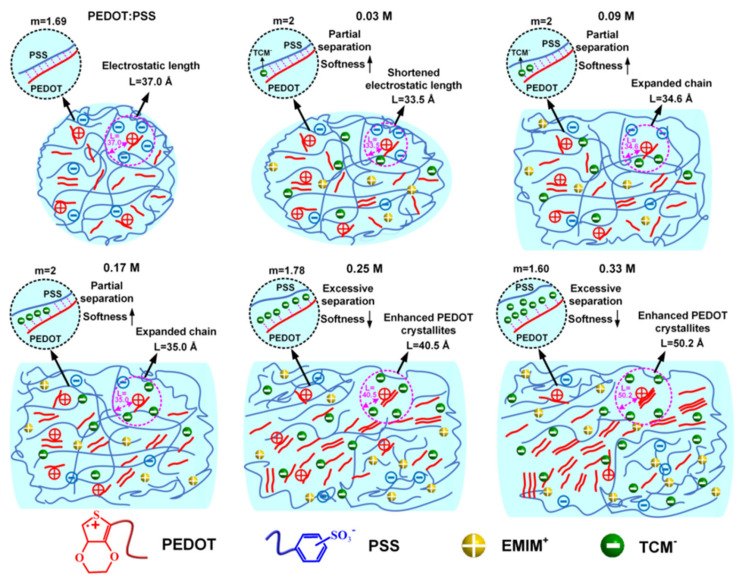
Schematic illustration of the supramolecular assembly of PEDOT:PSS/EMIM:TCM hybrids in aqueous solution as a function of EMIM:TCM content, revealed through combined SAXS and SANS analyses. Note: These arrows mark the structure features (*m* and *L* values) of the sample, where *m* reflects the stiffness of the polyelectrolyte chain, the higher the value, the softer of the chain and *L* is the electrostatic screening length of the interchain correlation, the higher the value, the larger the screening distance. Reproduced from Ref. [31], Springer Nature, 2022.

The chemistry of PEDOT:PSS allows extensive structural tuning. Variations in polymerization conditions, PSS-to-PEDOT ratio, and post-treatment solvents (such as dimethyl sulfoxide, ethylene glycol, or sulfuric acid) yield conductivities ranging from 0.1 to >1000 S cm^−1^ (Figure 3) [30,34]. The addition of secondary dopants reorganizes the polymer chains, enhancing π–π stacking and phase separation, while crosslinking agents such as (3-Glycidyloxypropyl) trimethoxy silane (GOPS) or divinyl sulfone improve film integrity and aqueous stability [35]. These tunable characteristics have made PEDOT:PSS the gold standard among solution-processable organic conductors (Figure 4) [31]. Importantly, these processing strategies not only optimize electrical performance but also control residual species-an issue later echoed in neural applications, where chemical purity and biocompatibility become critical [9,10,26].

In flexible electronics, PEDOT:PSS’s mechanical compliance and adhesive versatility have been equally important. Its low modulus (0.1–2 GPa) allows seamless integration with stretchable substrates and polymer matrices, while its hydrophilicity enables uniform deposition onto diverse surfaces, from plastics to textiles [6,28]. This ability to conform to soft, dynamic interfaces foreshadows its relevance to biological systems, where mechanical mismatch and poor adhesion often lead to device failure [36]. In fact, the same traits that permit PEDOT:PSS to sustain repeated bending and deformation in wearable electronics directly translate to the demands of chronic neural implantation [6,37].

The structure–property relationships established in the context of flexible electronics provide valuable insights into its performance at biological interfaces. For example, nanoscale phase separation between PEDOT-rich and PSS-rich domains dictates charge carrier pathways and water permeability-parameters that are directly tied to ionic transport and protein adsorption in physiological environments [22,24,30,38]. Similarly, control over film thickness and microstructure, achieved through additive-assisted deposition or vapor phase polymerization, affects the diffusion of ions and metabolites through the polymer, determining the stability of electrode performance over time [9]. Understanding these correlations enables the rational transfer of processing strategies from flexible device engineering to the design of stable, bioactive neural interfaces. Moreover, the conceptual understanding of PEDOT:PSS as an organic mixed ionic-electronic conductor (OMIEC) is grounded in comprehensive frameworks developed for organic bioelectronics and soft electronic materials. In particular, the reviews by Rivnay et al. [39] and Crispin et al. [40] have systematically clarified how coupled ionic and electronic transport emerges from polymer structure, morphology, and interfacial chemistry, providing a unifying basis for both electronic and bioelectronic device design.

However, when PEDOT:PSS transitions from inert device architectures to the complex neural milieu, its function extends beyond charge conduction [9,10]. In the latter context, the polymer no longer acts merely as a film or coating-it becomes the functional interphase that mediates electrochemical exchange and biological communication. The flexible electronics community has already demonstrated how secondary dopants, nanostructuring, and hybridization can dramatically influence interfacial transport and durability [23,33,37]. These lessons provide a blueprint for the neural interface community: the optimization of PEDOT:PSS microstructure, dopant chemistry, and post-processing can be leveraged not only to enhance conductivity but also to build selective, adaptive, and protective interphases akin to a bio-SEI.

Therefore, the chemical and mechanical success of PEDOT:PSS in flexible electronics should not be viewed as an endpoint, but rather as the foundation for its next transformation. The same mechanisms that control phase separation, dopant distribution, and chain ordering in films now underpin the control of ion transport, residue sequestration, and biostability in neural environments. By tracing this continuum—from organic conductors to bio-interphases—PEDOT:PSS emerges as a material platform uniquely positioned to serve as the functional SEI of neural probes, providing both electrical performance and biological compatibility.

## 3. The Unique Neural and Implantable Environment

Translating materials originally developed for flexible electronics into the neural interface domain exposes them to an environment of unprecedented chemical and biological complexity. Unlike the controlled conditions of dry or encapsulated devices, implanted electrodes operate within a dynamic milieu dominated by ions, proteins, enzymes, and reactive oxygen species [41]. The brain and peripheral nervous system are not static substrates—they are living, adaptive tissues whose molecular composition and mechanical state evolve over time [5,9,42]. In this setting, the electrode–tissue interface becomes the site of continuous exchange and adaptation, where the stability and functionality of the implanted material depend on its ability to coexist with the surrounding biological environment. Recent studies have revealed that organic conductors, including PEDOT:PSS, may undergo unintended electrochemical side reactions when simultaneously exposed to oxygen and water under physiological conditions [43,44]. These reactions can lead to the generation of reactive oxygen species (ROS), which not only degrade the electrical performance of the polymer but may also pose risks to surrounding neural tissue [45]. Such processes have been directly observed during device operation and electrical biasing, underscoring the importance of considering interfacial chemical stability in bioelectronic design [46].

**Figure 5:**
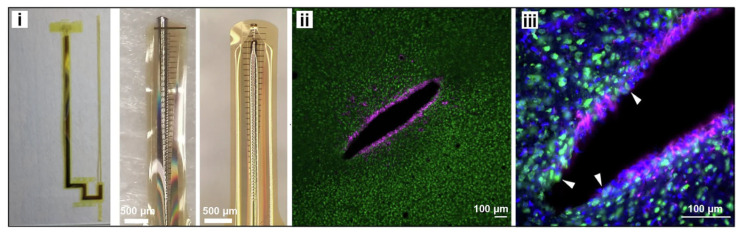
128-channel open-sheath flexible electrode and corresponding immunofluorescence response of GFAP and NeuN two weeks post-implantation, including (**i**) photo image of the electrode and tip, (**ii**) overall immunofluorescence response, and (**iii**) enlarged view of the immunofluorescence. Reproduced from Ref. [48], Springer Nature, 2025.

The neural microenvironment presents several specific challenges. Ionic strength and composition fluctuate across synaptic activity and extracellular signaling, involving gradients of Na^+^, K^+^, Ca^2+^, and Cl^−^ that can modulate the electrochemical potential at the electrode surface [47]. Proteins such as albumin, fibrinogen, and complement components rapidly adsorb onto foreign surfaces, initiating the foreign body response (Figure 5) [48]. Simultaneously, microglial activation and fibrotic encapsulation progressively increase interfacial impedance, reducing recording quality and stimulation efficiency. Mechanical motion caused by pulsatile blood flow or tissue micromotion further aggravates the interface, leading to microdelamination and accelerated degradation of polymer films. In such a setting, long-term device survival is governed not merely by bulk material stability but by the chemical and structural resilience of the interfacial layer that directly contacts the tissue.

For PEDOT:PSS, these challenges redefine its function. Rather than serving only as a conductive coating to improve electrode impedance, PEDOT:PSS becomes the bio-interphase responsible for maintaining selective and stable communication between the electronic probe and neural tissue [1]. The polymer’s intrinsic mixed conduction and hydrated morphology make it uniquely suited for this role. Its PSS domains absorb and exchange ions, allowing capacitive charge transfer, while the PEDOT-rich network ensures efficient electron transport to the underlying metallic substrate [36]. More importantly, the soft, hydrated character of the polymer mitigates mechanical stress and reduces cellular irritation-attributes that are rarely achieved simultaneously in inorganic system. For example, mechanical stiffness mismatch between neural tissue (typically in the kPa range) and implanted electrodes (often several orders of magnitude stiffer) leads to localized strain accumulation at the tissue-electrode interface [6,37]. Although PEDOT:PSS coatings are significantly softer than metal electrodes, their effective modulus increases upon dehydration or excessive crosslinking. Such stiffness variations can influence local contact stability and exacerbate micromotion-induced interfacial stress. Moreover, micromotion arising from respiration, pulsatile blood flow, and head movement induces cyclic shear and tensile stresses at the interface [49]. In PEDOT:PSS-based electrodes, such stresses may disrupt the microphase-separated morphology, alter hydration levels in PSS-rich domains, and modify ionic transport pathways. These mechanically induced changes can manifest as gradual increases in impedance, drift in electrochemical response, and loss of recording stability over chronic implantation.

However, the same properties that confer high ionic permeability can also lead to uncontrolled chemical exchange. Residual oxidants, acid traces, or secondary dopants from synthesis and post-treatment can leach into the surrounding medium, potentially disturbing the local pH or triggering cellular responses. Conversely, adsorption of biomolecules and infiltration of enzymes may alter the redox state of PEDOT, degrading its conductivity over time [50]. These bidirectional reactions echo the degradation phenomena observed in electrochemical energy systems, where electrolyte decomposition or interfacial instability necessitates the formation of a solid–electrolyte interphase (SEI) [19,51]. The analogy is instructive: in batteries, the SEI forms spontaneously to create a chemically selective yet ionically conductive barrier that protects both the electrode and the electrolyte [17,52,53]. Similarly, in neural implants, an engineered interphase with comparable selectivity and stability could preserve device function while maintaining tissue health.

From this perspective, PEDOT:PSS naturally acts as a biological analog of the SEI- a “bio-SEI” that selectively mediates ion transport and minimizes adverse chemical reactions. Its dual-phase structure inherently separates electronic and ionic pathways, while its tunable chemistry allows the introduction of stabilizing or bioactive components that control interfacial interactions. Indeed, several recent studies have shown that functionalizing PEDOT:PSS with zwitterionic, polydopamine, or hydrogel-based layers further enhances biocompatibility and reduces fouling, suggesting that a self-regulating, SEI-like interphase is both achievable and effective [11,54]. Yet, unlike the SEI in batteries, which forms spontaneously through reductive decomposition, the bio-SEI must be deliberately designed and pre-formed to ensure chemical purity and long-term stability within the physiological milieu.

Recognizing the neural environment as a chemically active, self-healing, and mechanically dynamic system fundamentally changes the design philosophy for electrode materials. In such an environment, the polymer is not simply an electrode modifier but an integral part of the biological interface. PEDOT:PSS, through its dynamic structure and responsive conduction, can be tailored to accommodate the fluctuating conditions of ionic concentration, enzymatic activity, and immune signaling. Conceptually, the ideal PEDOT:PSS bio-SEI would be ion-permeable but residue-impermeable, soft yet mechanically anchored, and electronically active yet biologically inert. Designing such an interphase demands a synthesis strategy that goes beyond maximizing conductivity to incorporate principles of selectivity, adaptability, and self-protection.

In summary, the neural and implantable environment transforms PEDOT:PSS from a functional coating into the core mediator of the electrode-tissue relationship. It is within this chemically and biologically reactive space that the bio-SEI concept takes on its full meaning: the polymer becomes not only a conduit for charge but also a shield, a buffer, and an adaptive layer that sustains long-term integration of electronics within living tissue.

## 4. Molecular and Structural Design of PEDOT:PSS for Neural Interfaces

The performance and reliability of PEDOT:PSS in neural interfaces depend fundamentally on its chemical structure and the purity of its synthesis. When PEDOT:PSS is viewed not merely as a conductive polymer but as a bio-solid–electrolyte interphase (bio-SEI), its design objectives shift from maximizing conductivity to achieving selective transport, chemical stability, and biocompatible durability. Every stage of its preparation-from oxidative polymerization to post-treatment-contributes to the chemical composition and functionality of this interphase.

### 4.1. Molecular Engineering and Control of Chemical Purity

PEDOT:PSS is typically synthesized through oxidative polymerization of 3,4-ethylenedioxythiophene (EDOT) in the presence of poly(styrenesulfonate) (PSS), which serves both as a charge-balancing dopant and a colloidal stabilizer [34]. The resulting dispersion consists of PEDOT-rich grains enveloped by PSS chains, whose relative composition and organization dictate the balance between conductivity and hydrophilicity. Controlling the stoichiometry and oxidation level is crucial because residual oxidants such as ferric ions or persulfates can compromise cell viability and accelerate electrochemical degradation. Experimental evidence for residual oxidants and counterions in PEDOT:PSS has been obtained using techniques such as X-ray photoelectron spectroscopy, ion chromatography, elemental analysis, and electrochemical measurements. For example, sulfate and persulfate residues have been detected after polymerization, while excess PSS has been shown to dominate surface composition and ion exchange behavior. These findings underscore that chemical purity in PEDOT:PSS films is relative rather than absolute, and strongly dependent on synthesis route and post-treatment [23]. Recent progress in enzyme-assisted polymerization and electrochemical growth under physiological conditions has enabled cleaner synthesis pathways, producing films with minimal residues and enhanced biocompatibility [55].

### 4.2. Post-Treatment and Reactive Functionalization

Beyond synthesis, post-treatments play a decisive role in defining the functional structure of PEDOT:PSS [23]. Secondary dopants-ethylene glycol, DMSO, ionic liquids, or zwitterionic compounds-promote chain rearrangement and domain alignment, but they also alter the local chemical landscape. Some residues, though beneficial for conductivity, can persist as potential cytotoxins or interfere with the host immune system [56]. This challenge has led to the introduction of reactive purification strategies that simultaneously remove residual reactants and introduce functional surface groups. For example, treatment with catechol-based molecules such as dopamine or tannic acid forms ultrathin polydopamine (PDA) coatings that scavenge metal impurities, crosslink with PSS sulfonates, and endow the surface with intrinsic adhesion and antioxidative capacity [57,58]. The resulting interface bears resemblance to a biological “protective skin,” capable of stabilizing the underlying polymer against oxidative stress and mechanical fatigue.

### 4.3. Mesoscale Morphology and Transport Pathways

At the mesoscale, the morphology of PEDOT:PSS reveals phase-separated domains of conductive PEDOT embedded within a less conductive PSS matrix. The continuity and orientation of these domains govern the transport of both electrons and ions, establishing the foundation of the mixed conduction behavior critical for neural communication [9]. Solvent-assisted phase rearrangement can induce vertical stratification-an internal gradient in conductivity and hydrophilicity that favors the formation of a stable outer shell [21,22]. In this way, structural design serves as a precursor to interfacial selectivity, much as in the formation of the solid–electrolyte interphase (SEI) in batteries. Moreover, from an interfacial perspective, ROS generation can be viewed as a manifestation of insufficient passivation at the polymer–electrolyte boundary [43,44]. This provides a strong rationale for introducing SEI-inspired protective interphases (bio-SEI), which are designed to be ion-permeable while suppressing oxygen- and water-driven redox reactions. Such layers may therefore decouple electrical functionality from chemical degradation and biological risk.

### 4.4. Formation of SEI-like Protective Interphases

**Figure 6:**
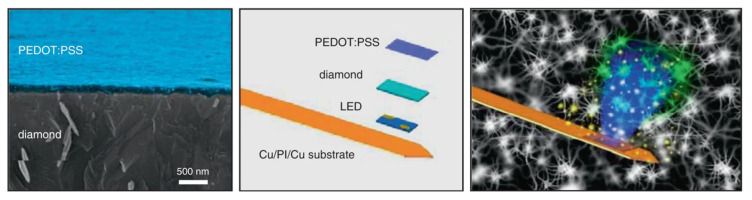
Cross-sectional SEM image of a PEDOT:PSS film (colorized in blue) on diamond, alongside an exploded schematic of the microprobe comprising a blue micro-LED, diamond interlayer, and PEDOT:PSS thin film on Cu/PI/Cu substrate, and a conceptual illustration of the microprobe system for optogenetic stimulation and dopamine detection. Reproduced from Ref. [59], Springer Nature, 2020.

Drawing inspiration from SEI chemistry, researchers have begun to conceptualize a bio-SEI surrounding PEDOT:PSS electrodes: an intentionally formed, ion-permeable, and biocompatible layer that decouples charge transport from direct biochemical contact (Figure 6) [59]. Unlike inert encapsulation layers, this interphase remains conductive to essential ions while blocking undesirable molecular infiltration and oxidative agents [60]. Thin coatings of PDA, PEGylated polymers, or zwitterionic copolymers have been demonstrated to reduce protein fouling and inflammatory cell adhesion without impeding ionic conduction [57,58]. In some cases, hydrogel layers rich in hydrogen-bonded or boronate linkages have provided partial self-healing capability, allowing the interface to recover from minor mechanical disruption-an essential trait for long-term implants subjected to micromotions [49]. Moreover, to contextualize the role of PEDOT:PSS as a bio-interphase material, it is instructive to compare it with other classes of interfaces widely used in neural and implantable bioelectronics, including inert polymer coatings, conductive hydrogels, metal-based surface modifications, and zwitterionic antifouling layers [2,61,62,63,64,65,66]. While each class addresses specific interfacial challenges, PEDOT:PSS uniquely combines mixed ionic-electronic transport with mechanical compliance and processability, positioning it between passive encapsulation layers and purely electronic conductors (Table 1).

The long-term functionality demonstrated in large-scale neural systems, such as the Neuralink platform, Ref. [13] suggests that PEDOT:PSS effectively forms a selective and adaptive interphase at the tissue-electrode boundary. From this perspective, the PEDOT:PSS layer can be viewed as a bio-SEI, which permits ionic signal transduction while suppressing detrimental chemical reactions and excessive inflammatory responses. Recent studies on PEDOT-based stretchable batteries further illustrate how conductive polymers can operate at electrochemical interfaces requiring simultaneous ionic transport, mechanical deformability, and interfacial stability. In particular, the work reported by Rahmanudin et al. Ref. [67] demonstrates that PEDOT-derived materials can sustain repeated electrochemical cycling when appropriate interfacial control is achieved. This behavior closely parallels the function of the solid–electrolyte interphase in lithium-ion batteries, reinforcing the relevance of SEI-inspired concepts for designing stable bioelectronic interfaces.

Notably, in contrast to the classical SEI in lithium batteries-which forms via electrolyte decomposition at highly reducing potentials-the proposed bio-SEI at PEDOT:PSS-tissue interfaces arises through gradual interfacial reorganization [14,17]. Driven by mild electrochemical bias, ionic exchange, and exposure to biological fluids, this formation represents an adaptive, dynamic process rather than a discrete phase transition.

### 4.5. Structure–Property–Function Correlation Across Hierarchies

The structural hierarchy of PEDOT:PSS, extending from molecular conformation to interfacial assembly, thus establishes a direct correlation between structure, function, and biological response [24,31]. Molecular design ensures chemical safety; mesoscale ordering governs transport efficiency; and nanoscale interphases maintain equilibrium with the biological environment. Together, these levels define a material system that transcends the role of a passive conductor, emerging instead as a dynamic mediator between synthetic electronics and living neural tissue.

## 5. Toward Interfacial Stability and Clinical Translation

As PEDOT:PSS transitions from research prototypes to clinically relevant bioelectronic devices, interfacial stability becomes the dominant design consideration. The challenge lies in preserving both electrical performance and biological tolerance over months or even years of operation. Traditional strategies that rely on high conductivity alone often fail under physiological conditions, where ionic gradients, enzymatic activity, and microglial responses continuously remodel the electrode-tissue boundary. The SEI-inspired framework offers a new perspective: long-term stability can be achieved not by inert isolation, but by the controlled formation of a selective, self-regulated interphase [56].

### 5.1. Hybrid Architectures for Mechanical and Ionic Compatibility

Hybrid and composite systems have proven particularly effective in realizing this concept. By incorporating PEDOT:PSS into hydrophilic polymer networks-such as poly(vinyl alcohol), polyethylene glycol, or naturally derived polysaccharides-researchers have created conductive hydrogels whose softness and water content closely mimic neural tissue [60]. These materials accommodate dynamic strain while maintaining ion transport, thereby reducing micromechanical mismatch and local inflammation [68]. Similarly, interpenetrating networks that combine PEDOT:PSS with elastomers such as polyurethane or PDMS extend mechanical durability and adhesion under cyclic deformation [69]. The addition of nanostructured fillers like graphene, MXenes, or gold nanoparticles further enhances charge transfer efficiency, provided that these conductive agents are passivated or coated to prevent cytotoxic interactions [54,70].

### 5.2. Chronic Biocompatibility and Electrochemical Durability

In vivo, the benefits of these hybrid architectures become evident through reduced impedance drift, improved signal-to-noise ratio, and attenuated glial encapsulation [71]. Longitudinal studies have demonstrated that ion-permeable coatings and hydrogel encapsulations can maintain electrochemical performance for several months in animal models, suggesting that properly engineered interphases can convert acute biocompatibility into chronic stability [36]. The concept of the bio-SEI naturally integrates with these findings: a soft yet selective interphase that modulates molecular exchange while suppressing the accumulation of reactive oxygen species and denatured proteins.

### 5.3. Convergence Toward Clinical and Commercial Platforms

From a translational standpoint, reproducibility and safety are as important as performance. The fabrication of PEDOT:PSS-based electrodes increasingly employs scalable methods such as inkjet, screen, or aerosol printing, enabling conformal coverage on flexible substrates with controlled film morphology and conductivity [37]. Recent advances in ink formulation allow the incorporation of functional additives-such as crosslinkers, dopants, or biocompatible binders-that can spontaneously reorganize during film formation to produce compositionally graded or passivated surfaces [72,73]. In addition, from a bioelectronic perspective, residual oxidants and mobile counterions may contribute to unintended redox reactions, local pH shifts, or oxidative stress in neural tissue [39]. These considerations have motivated recent efforts to minimize residual chemical species through alternative polymerization routes, extensive solvent exchange, or interfacial passivation strategies. In this context, the proposed bio-SEI concept offers a complementary approach, whereby a stabilized interphase may isolate residual species from direct interaction with the biological environment [40].

Rather than invoking a direct electrochemical analogy to the SEI in batteries, these surface-restructuring and self-passivating phenomena can be viewed as a conceptual parallel: they provide a thin, stable layer that mediates ionic and electronic transport while mitigating unwanted interfacial reactions. This “bio-interphase” concept offers a rational framework for designing protective yet ion-permeable coatings around conducting polymer electrodes. Importantly, such approaches align with existing biocompatibility and manufacturing standards-such as ISO 10993 for biological evaluation and FDA guidance on chemical characterization of medical devices -which emphasize control of extractables, residual reagents, and surface chemistry consistency across scalable fabrication routes [74].

## 6. Outlook and Conclusions

The development of PEDOT:PSS for neural interfaces mirrors a broader transformation in materials science: a shift from optimizing static performance toward mastering dynamic interfacial behavior [11]. The analogy to the SEI in batteries offers more than a conceptual link—it serves as a design principle. In electrochemical systems, stability is achieved when the interface becomes self-regulating, maintaining equilibrium between opposing chemical and mechanical forces. Translated to neural systems, this insight points toward materials that do not resist the biological environment but coexist with it, establishing a cooperative equilibrium between electronic function and living tissue.

In this framework, PEDOT:PSS functions as a bio-SEI that bridges electronic and biological domains. Its mixed ionic-electronic conduction, soft mechanics, and tunable chemistry allow it to mediate charge transfer while buffering chemical and mechanical fluctuations. Yet its future potential extends beyond its current use as a stable coating. The next step lies in designing PEDOT:PSS architectures that actively maintain interfacial homeostasis—responding to biochemical changes, repairing minor damage, and preserving electrochemical balance over years of operation [3,48]. This transition from passive durability to adaptive stability will define the next generation of biointegrated electronics [5,50].

Progress will rely on integrating molecular-level precision with system-level intelligence. Operando techniques, including synchrotron-based X-ray scattering, X-ray absorption fine structure, and in situ scanning probe microscopy, are beginning to reveal how ionic and electronic currents dynamically reshape PEDOT:PSS under physiological conditions [18,31]. From a practical perspective, measurable targets for next-generation PEDOT:PSS bio interface may include (a) maintaining stable ionic diffusivity within one order of magnitude over >10^6^ stimulation cycles, (b) minimizing hydration-induced volumetric strain below 5–10% under physiological conditions, and (c) suppressing long-term interfacial potential drift at the electrode–electrolyte boundary. These quantities can be directly linked to molecular descriptors such as ion migration barriers, water–polymer interaction energies, and local electrostatic field distributions. These insights can establish quantitative design rules for controlled bio-SEI formation and regeneration. Parallel advances in data-driven materials discovery-from machine learning-guided synthesis optimization to AI-assisted modeling of interfacial aging-will enable predictive design of polymer interfaces with programmable stability and adaptive feedback control [5,75]. Notably, recent advances in molecular dynamics, coarse-grained modeling, and machine-learning-assisted force-field optimization make it feasible to map these descriptors across large compositional and processing spaces [75]. For example, diffusion barriers and hydration free energies can be extracted from atomistic or coarse-grained simulations, while interfacial potential gradients may be inferred from coupled Poisson–Nernst–Planck models [75]. When combined with experimental feedback, such descriptors offer a rational basis for AI-assisted screening of stable and biocompatible PEDOT:PSS formulations.

As the technology moves closer to clinical and commercial realization, the ethical and environmental dimensions of material design must evolve alongside functional performance. Long-term neural interfaces demand absolute chemical transparency: residue-free synthesis, non-toxic degradation pathways, and recyclable device components. PEDOT:PSS, with its water-based processing and molecular tunability, already provides a favorable foundation [26]. Achieving truly inert and renewable interphases will consolidate its standing as the benchmark material for sustainable neural bioelectronics.

**Figure 7:**
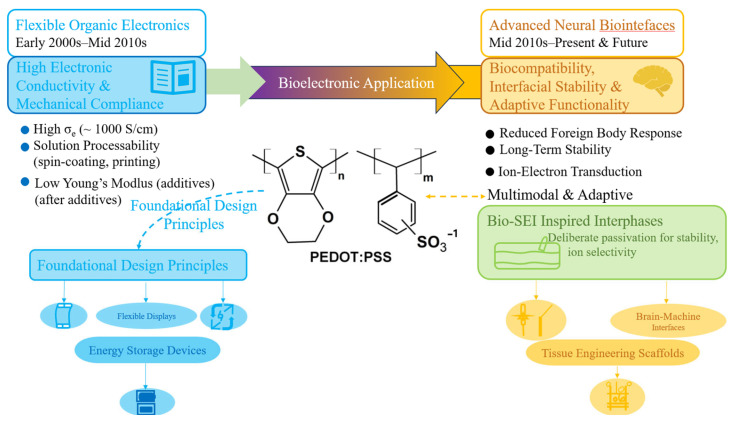
The future trajectory of PEDOT:PSS bioelectronics, emphasizing the ‘bio-SEI’ concept. The schematic illustrates a technological shift from high-conductivity flexible organic electronics (blue zone) toward biocompatible neural interfaces (orange zone). While early research prioritized electronic conductivity and mechanical compliance for displays and energy storage, and reduced foreign body responses. Central to this evolution is the versatile PEDOT:PSS molecular backbone, which enables the emerging "bio-SEI" framework (green node). This concept utilizes deliberate passivation and ion-selective layers to achieve the long-term interfacial stability and adaptive functionality required for chronic, seamless bio-integration, bridging foundational polymer processing with sophisticated implantable electronics.

Ultimately, PEDOT:PSS is more than a successful conductive polymer—it represents a conceptual archetype for the next era of bio-integrated electronics. The SEI analogy reframes interfacial design as a matter of dynamic governance rather than passive protection, guiding researchers to create materials that conduct not only charge but also communication between the synthetic and the biological (Figure 7). Just as the SEI transformed electrochemical energy storage from a fragile reaction boundary into a controllable design feature, the bio-SEI may define the future of stable, intelligent, and ethically grounded neural technologies. At this intersection of chemistry, biology, and computation, PEDOT:PSS stands as both a model system and a roadmap—demonstrating how matter itself can be engineered to negotiate coexistence between human physiology and electronic intelligence [58].

## Figures and Tables

**Table 1:** Comparison of Representative Bio-Interphase Materials for Neural Interfaces.

Bio- Interphase Material	Mixed Ionic– Electronic Transport	Mechanical Compliance	Chronic Biocompatibility	Interfacial Stability	Key Advantages	Key Limitations	Representative References
PEDOT:PSS	Yes	Moderate-high (tunable via hydration/hybrids)	Good (with surface control)	Moderate (ROS-sensitive)	Low impedance, signal amplification, scalable processing	ROS generation, chemical residue sensitivity	Rivnay *Nat. Rev. Mater.* 2018 [61] Khodagholy *Nat. Neurosci.* 2015 [62]
Conductive hydrogels	Yes (ionic-dominant)	Very high (kPa range)	Excellent	Moderate	Tissue-like mechanics, low inflammation	Low electronic conductivity, dehydration	Yuk *Nat. Mater.* 2016 [63] Yang *Nat. Rev. Mater.* 2018 [64]
Parylene C	No (insulating)	Low	Excellent	High	Robust encapsulation, FDA approved	High impedance, signal attenuation	Seymour *Biomaterials* 2007 [65]
Metal oxides (IrOx, TiN)	Electronic only	Very low	Good	High	High charge injection capacity	Mechanical mismatch, delamination	Cogan *Annu. Rev. Biomed. Eng.* 2008 [2]
Zwitterionic polymer layers	No (passive)	Moderate	Excellent	High	Antifouling, protein resistance	No signal transduction	Jiang *Adv. Mater.* 2010 [66]

## Data Availability

No new data were created or analyzed in this study.
